# Structural Insight of KSIII (β-Ketoacyl-ACP Synthase)-like Acyltransferase ChlB3 in the Biosynthesis of Chlorothricin

**DOI:** 10.3390/molecules27196405

**Published:** 2022-09-28

**Authors:** Asad Ullah Saeed, Mueed Ur Rahman, Hai-Feng Chen, Jianting Zheng

**Affiliations:** 1 State Key Laboratory of Microbial Metabolism and School of Life Sciences and Biotechnology, Shanghai Jiao Tong University, Shanghai 200240, China; 2 State Key Laboratory of Microbial Metabolism, Joint International Research Laboratory of Metabolic & Developmental Sciences, Department of Bioinformatics and Biostatistics, National Experimental Teaching Center for Life Sciences and Biotechnology, School of Life Sciences and Biotechnology, Shanghai Jiao Tong University, Shanghai 200240, China; 3 Shanghai Center for Bioinformation Technology, Shanghai 200235, China; 4 Joint International Research Laboratory of Metabolic & Developmental Sciences, Shanghai Jiao Tong University, Shanghai 200240, China

**Keywords:** acyltransferase, ChlB3, hydrolysis, catalytic efficiency, structural insight

## Abstract

Chlorothricin (CHL) belongs to a spirotetronate antibiotic family produced by *Streptomyces antibioticus* that inhibits pyruvate carboxylase and malate dehydrogenase. For the biosynthesis of CHL, ChlB3 plays a crucial role by introducing the 6-methylsalicylic acid (6MSA) moiety to ChlB2, an acyl carrier protein (ACP). However, the structural insight and catalytic mechanism of ChlB3 was unclear. In the current study, the crystal structure of ChlB3 was solved at 3.1 Å-resolution and a catalytic mechanism was proposed on the basis of conserved residues of structurally related enzymes. ChlB3 is a dimer having the same active sites as CerJ (a structural homologous enzyme) and uses a KSIII-like fold to work as an acyltransferase. The relaxed substrate specificity of ChlB3 was defined by its catalytic efficiencies (*k*_cat_/*K*_m_) for non-ACP tethered synthetic substrates such as 6MSA-SNAC, acetyl-SNAC, and cyclohexonyl-SNAC. ChlB3 successfully detached the 6MSA moiety from 6MSA-SNAC substrate and this hydrolytic activity demonstrated that ChlB3 has the potential to catalyze non-ACP tethered substrates. Structural comparison indicated that ChlB3 belongs to FabH family and showed 0.6–2.5 Å root mean square deviation (RMSD) with structural homologous enzymes. Molecular docking and dynamics simulations were implemented to understand substrate active site and structural behavior such as the open and closed conformation of the ChlB3 protein. The resultant catalytic and substrate recognition mechanism suggested that ChlB3 has the potential to use non-native substrates and minimize the labor of expressing ACP protein. This versatile acyltransferase activity may pave the way for manufacturing CHL variants and may help to hydrolyze several thioester-based compounds.

## 1. Introduction

Spirotetronate antibiotics have different biological activities and distinctive architectures [1]. They contain a unique aglycone that comprises tetronic-acid (spiro connected to a cyclohexene ring) conjugated to a carbonyl group (e.g., kijanimicin) or by carboxylic ester via a *trans* decalin system (e.g., chlorothricin, CHL) [2]. Furthermore, amendment of the structurally associated aglycones, with peripheral moieties or by a variety of deoxy sugars, represents the diversity of potential structures [2]. CHL is the first spirotetronate family member whose structural details have been determined and produced by *Streptomyces antibioticus* DSM 40725 [1,3]. The CHL gene cluster comprises 122 kb of contiguous DNA and has 42 open reading frames, of which 35 are related to CHL biosynthesis [2]. CHL consists of chlorothricolide and two D-Olivose saccharides with a 3′ hydroxyl position related to secondary sugar adorned by a 2-methoxy-5-chloro-6-methylsalicyclic acid moiety, which is essential for the stability and antibacterial activity of CHL [4,5,6].

**Figure 1:**
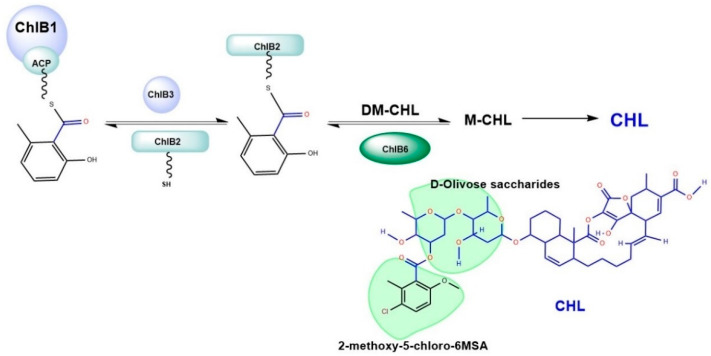
An overview of CHL biosynthesis. ChlB3 helps transfer of 6MSA from ChlB1 to holo-ChlB2, resulting in 6MSA-S-ChlB2. Following that, ChlB6 immediately grafts holo-6-methylsalicyl ChlB2 onto DM-CHL to produce M-CHL.

6-methylsalicyclic acid (6MSA) moiety is synthesize by ChlB1 (an iterative type-I PKS) from one acetyl- and three malonyl-CoA [5,7,8]. However, 6MSA produced by ChlB1 is not released from the pantetheinyl group due to the absence of thioesterase [9]. Incorporation of 6MSA moiety into CHL depends upon the ChlB3 that helps transfer the 6MSA from ChlB1 to holo-ChlB2, resulting in 6MSA-S-ChlB2. Afterwards, holo-6-methylsalicyl ChlB2 is instantly grafted by ChlB6 onto DM-CHL to create M-CHL. The last step is for ChlB4 and ChlB5 to chlorinate and O-methylate M-CHL to make DCM-CHL or CHL, Figure 1 [9]. According to functions and architecture, polyketide synthases (PKSs) are categorized into various types [10]. The type I polyketide synthases (type I PKSs) are large, multifunctional proteins with several modules and domains that carry out a specific enzymatic process. Each module is in charge of carrying out a single condensation cycle in a non-iterative manner. This system is also known as a modular PKS since it utilizes several modules. Each module contains the crucial domains acyltransferase, ketosynthase, and acyl carrier protein (ACP), which work together to create the intermediate -keto ester. Keto group modification is carried out by the enzymes -ketoreductase, dehydratase, and enoyl reductase, which are additional domains that might be included in the module [11,12]. Whereas type II PKSs are made up of freestanding elements that form loose complexes. Enzymes belonging to the chalcone synthase superfamily of biocatalysts are called type III PKSs. These enzymes have multiple functions [13,14,15].

It has been stated that ChlB3 works in a time-dependent manner, representing the conversion of 6MSA-S-ChlB1 to 6MSA-S-ChlB2 with an activity of 7.33 min^−1^ [9]. ChlB3 has also been shown to catalyze the transfer of 2-methoxy-5-chloro-6-methylsalicyl and 2-methoxy-6-methylsalicyl moieties with 0.008 min^−1^ and 0.006 min^−1^ activities, respectively. In light of previous studies, different ACP tethered substrates could be catalyzed by ChlB3. Furthermore, the specificity of ChlB3 for different substrates represents the variable tolerance of the ChlB3 enzyme [9]. Functional analysis of ChlB3 revealed that production of acyl-S-ACP, as an active substrate, was the last attachment step. However, structural characterization and catalytic mechanisms of ChlB3 still need to be addressed.

The sequence of ChlB3 is related to ketoacylsynthases III (KS III) homologous (DpsC and CerJ). Except for Cerj, which transfers the malonyl unit onto a sugar hydroxyl residue, all of these enzymes catalyzed claisen condensation [9,16]. As part of diverse fatty acid synthases (FAS) and PKS, the functionally similar KS III is known to catalyse the initial elongation step in poly-β-keto processing. All ketosynthases share the thiolase fold and catalyse the synthesis of C-C bonds using acyl and malonyl building blocks [17,18]. Herein, we report the crystal structure of ChlB3 that provides molecular insight to understand the catalytic mechanism for 6MSA-SNAC, cyclohexonyl-SNAC, and acetyl-SNAC. The work also included a functional analysis to identify the distinct feature of ChlB3 that allows it to catalyze non-ACP-tethered substrates. Molecular dynamic (MD) simulations helped to investigate the substrate recognition residues as well as the conformational changes in structure. Finally, this study helped to understand the catalytic mechanism for thioester-based compounds that might be used in synthesizing novel antibiotics.

## 2. Results and Discussion

### 2.1. Overall Structure

**Figure 2:**
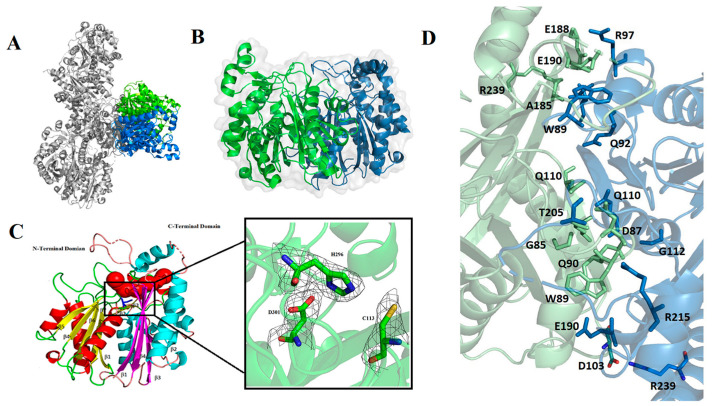
An overview of ChlB3 structure with an active site pocket and conserved residues (**A**) structure of ChlB3 comprising eight monomers. (**B**) surface view of dimeric organization of ChlB3 (**C**) Structure containing αββ(β)αββ motifs distributed in two equal parts. The first motif is represented in yellow and red, and second motif is represented in cyan and magenta. Amino acid residues belonging to the c-terminal region are displayed in wheat color and active site pocket is represented in red color, second portion represent the 2Fo-Fc electron density map of ChlB3 conserved residues contoured at 1.0 σ (**D**) A zoomed-in view of the dimeric interface of ChlB3.

The protein structure was present in the space group of P2_1_2_1_2_1_ with four dimers per asymmetric unit (Figure 2A). The refined model of ChlB3 contained 334 residues in each monomer and no electron density was observed for its 197–201 and 227–230 residues. The molecular weight of ChlB3 was confirmed by SDS-PAGE (Figure S1). The residue percentage in the most favored and allowed regions in the Ramachandran plot are depicted in Figure S2. ChlB3 structure is organized as a homodimer and each monomer having its own active site (Figure 2B). Each ChlB3 monomer has a seven-layered core composed of two layers of helices interspersed by five layers of sheet, with several connecting loop regions around the core. The core region features an internal duplication of two segments (14–183 and 184–339) that are comparable in structure except at their loop regions (Figure 2C), although there is no major sequence similarity between the two halves. A total of 2627.6 Å area is buried in the dimer interface of ChlB3 protein. This buried area is separated into polar and nonpolar contributions. The main chains of Thr205, Arg215, Gly112, Ala185, Arg239, Gln110, Gln92, Gln90, Gly102, Asp103, Trp89, Gly85 and Glu190 are involved in hydrogen bonding at the monomer-monomer interface. Other intermolecular interactions of different monomers include salt bridges present between Arg239, Asp103 (Figure 2D). The structure of ChlB3 indicates that the above-mentioned residues in the dimer interface may play a significant role in dimer formation.

DALI server confirmed the overall structure of ChlB3 resembles with ketoacylsynthases (KSIII) homologous CerJ (Protein Data Bank (PDB) code-3S3L, 0.84 Å RMSD) and Doxorubicin synthase-DpsC (5WGC, 0.6 Å RMSD) [9,16,19]. Sequence identity among these structurally related proteins is 32.48% and 34.50%, respectively. Notably, all these enzymes catalyzed claisen-condensation except Cerj, which transfers the malonyl unit onto a sugar hydroxyl residue. The superposition of ChlB3 with the structurally related CerJ protein revealed a structural deviation in the C-terminal region (248–339 residues) of each monomer (shown in orange in Figure 2C). Remarkably, all the structurally related enzymes share a similar active side pocket to catalyze the reaction (Figure S3).

### 2.2. The Structure of ChlB3 Resembles a KS-III like Fold

**Figure 3:**
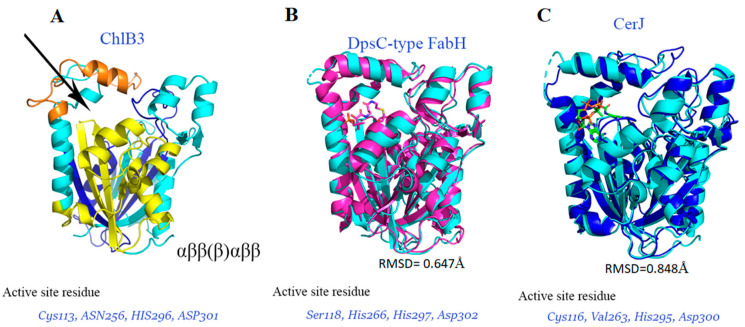
Comparison of the ChlB3 structure with homologous enzymes (**A**) ChlB3 structure with conserved active site residues (**B**) A superimposed structure of a structural homologous enzyme (DpsC-type FabH) exhibits the conserved active site residues. (**C**) ChlB3 was superimposed with structurally homologous enzyme CerJ, revealing the conserved residues coherent with ChlB3.

In light of phylogenetic analysis and the homologous enzymes, ChlB3 is found as acyltransferase. According to the cladogram, ChlB3 is closely associated with KSIII homologous (DpsC and CerJ) (Figure S4). Whereas the detailed examination of the amino acid sequence alignment and threading of the ChlB3 amino acid sequence onto the structurally related DpsC revealed that ChlB3 differs in region containing the highly conserved catalytic triad Ser-His-His (Figure 3 and Figure S5). In DpsC enzyme (Ser-His-His), side chains of histidine do not significantly improve the nucleophilic character of the catalytic cysteine, but His266-His297 point to the same place and function as hydrogen bond donors to stabilize the enolization phase [20,21]. In contrast, His296 directly interacts with Cys113 in ChlB3 to significantly boost its nucleophilicity. As a result, the His296-Asp301 pair increases Cys113 reactivity, which is also found in the KS III homologous CerJ protein. Generally, in KSIII enzymes cystine is responsible for the transacylation process, although histidine and aspartic acid catalytic residues crucial for Claisen-like condensation [22]. ChlB3 catalytic triad (Cys113, His296 and Asp301) as shown in Figure 2C, significantly differs in electrostatic character and geometry, despite the catalytic cysteine (Cys113) occupying the same position as those seen in both structurally related enzymes (Figure 3).

Previously, He et al., revealed that C113A mutant completely abolish acyl-transfer activity in ChlB3 [6]. Regarding the activity of these conserved residues in CerJ, only the D301A mutant reserved activity (0.3%) to the wild-type protein. However, the remaining mutants C116S, C116A, and H295N showed complete abrogation of malonyl transfer activity that highlighted the importance of these conserved residues [22]. Therefore, this catalytic triad (Cys113, His296 and Asp301) is highly influenced during the catalyzing reaction of the substrate.

### 2.3. Catalytic Mechanism of ChlB3

**Figure 4:**
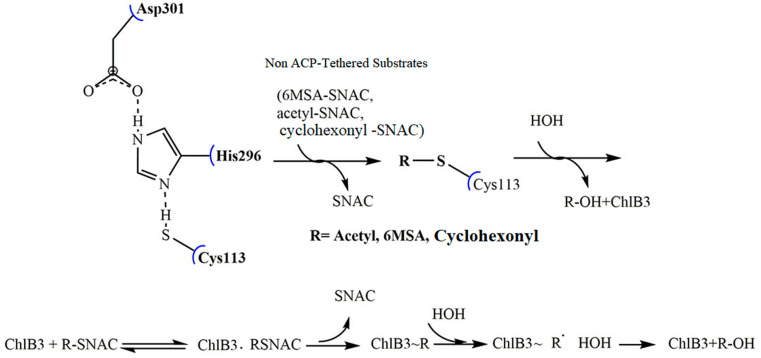
Model of ChlB3 ping-pong bi-bi mechanism. Reaction was initiated by introduction of substrates (6MSA-SNAC, acetyl-SNAC and cyclohexonyl-SNAC) into the active site of protein. Conserved catalytic residues Cys113, His296 and Asp301 hydrolyze the substrate and release the SNAC from R-SNAC.

Like ChlB3, many acyltransferases have been identified in various natural product, i.e., Caliheaicin, Polyketomycin, Maduropeptin, Coumermycin-A1, Pactamycin, Benzoxazole, and cervimycin that responsible for the formation of C-N, C-O and C-S bonds with the similar mechanism of C-S bond formation catalyzed by malonyl-CoA acyl carrier protein transacylases (FabD) [9,23]. These acyltransferases transfer an acyl group from CoA to ACP by a ping-pong bi-bi mechanism [24]. A similar mechanism was observed in ChlB3 that catalyzed a group of substrates, i.e., (6MSA-SNAC, cyclohexonyl-SNAC, and acetyl-SNAC), as shown in Figure 4. According to the adopted model, Cys113 nucleophile at the terminal of the substrate channel is triggered by His296. It captured hydrogen from sulfhydryl and maintained the positive influence of the dipole moment of *α*5. Consequently, adjacent Asp301 stabilized the fundamental character of His296. The reaction was started by introducing the 6MSA-ChlB1 into ChlB3, preceded by nucleophilic attack of Cys113 thiol. Eventually, the 6MSA moiety was loaded on to Cys113. The 6MSA moiety is taken up by the holo-ChlB2 from Cys113. Due to the lack of holo-ACP, enzyme intermediate (6MSA-SNAC) is promptly hydrolyzed by the water molecule [25].

### 2.4. Specificity in Hydrolytic Reactions

**Figure 5:**
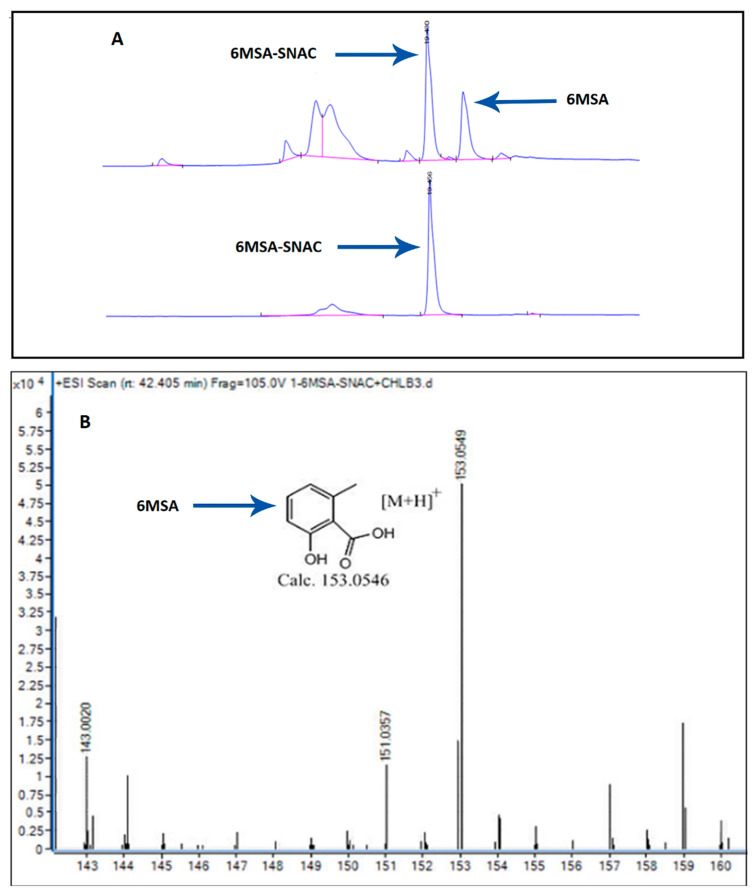
Chromatographic analysis of 6MSA produced from 6MSA-SNAC in response to hydrolysis. (**A**) HPLC profile showed the production of 6MSA as compared to standard 6MSA-SNAC. A single higher peak indicated the control and the hydrolyzed 6MSA was exhibited with smaller peak. (**B**) The observed result was labeled on mass spectra.

A previous study reported that ChlB3 is involved in the conversion of 6-methylsalicyl-S-ChlB1-ACP to 6-methylsalicyl-S-ChlB2 with catalytic efficiency *k*_cat_/*K*_m_ = 1690.70 mM^−1^ min^−1^ [9]. ChlB3 also has the ability to transfer 5-chloro-6-methyl-O-methylsalicyl-S-ChlB1 to 5-chloro-6-methyl-O-methylsalicyl-S-ChlB2 with catalytic efficiency *k*_cat_/*K*_m_ = 0.45 mM^−1^ min^−1^ [9]. Thus, covalent tethering of acyl-S-ACP is a general approach to sequestering the substrate. In the literature, it is also reported that acyl-SNAC thioesters can be incorporated into polyketide synthase [26]. We intended to evaluate the hydrolytic potential of ChlB3 against non-native acyl-SNAC (mimics the phosphopantetheinyl arm) thioesters instead of the acyl-S-ACP substrate [27]. The capability of ChlB3 to hydrolyze a panel of three synthetic acyl-SNAC thioesters was measured by using Ellman’s reagent, a common method to assess the SH groups in a protein [21]. Spectrophotometer was employed to analyze free SH and released SNAC [21]. Control reactions were also accomplished in parallel to ensure the hydrolytic activity of enzyme towards acyl-SNAC. For 6MSA-SNAC, acetyl-SNAC, and cyclohexonyl-SNAC, the steady-state kinetic parameters, (*k*_cat_/*K*_m_), were employed i.e., 25.7 ± 0.103 mM^−1^ min^−1^, 13.69 ± 0.240 mM^−1^ min^−1^, and 14.63 ± 0.26 mM^−1^ min^−1^ respectively. To ensure the correct hydrolytic activity of enzyme, the product (6MSA) was confirmed by mass spectrum (expected 153.0546, observed 153.0549) under positive ion mode, as shown in Figure 5 and Figure S6.

According to the kinetic parameters, the observed catalytic efficiency for 6MSA-SNAC was about 1.8-fold higher than acetyl-SNAC. ChlB3 preferred to catalyze substrates containing benzene group i.e., (6MSA) but showed slightly less activity towards non-benzyl substrate (cyclohexonyl). It might be due to lack of hydrophobic interactions of non-benzyl group with active site residue. The 6MSA moiety is involved in hydrogen bonding and hence stabilize the substrate in active site of ChlB3, as shown in snapshot of average structure extracted from 200 ns of MD simulation (Figure S7). However, in the case of cyclohexonyl-SNAC, the lack of an OH group makes the substrate unstable and lowers the catalytic efficiency. Collectively, these results suggest that ChlB3 could function as an acyltransferase with relaxed specificity, accepting acyl-SNAC thioesters in vitro without excessive use of ACP protein.

Active sites play a crucial role in the catalysis, stabilize the intermediates and enable the substrate to form enough contact points for strong binding [28]. ChlB3 crystals were soaked in the mother liquid comprising 4 mM 6MSA-SNAC; however, the corresponding electron density was not observed. To investigate the active site residues, 6MSA-SNAC was introduced into the active site of protein manually using coot program (version 0.9.8.1). A CerJ complex with CoA (3T6S) was considered to guide the modeling [22]. The substrate (6MSA-SNAC) was placed into the deep cavity whereas the SNAC portion was modeled towards the entrance. The modeled results clarified that 6MSA-SNAC was accepted by ChlB3 in respective active site. The modeling of substrate revealed the 14 residues i.e., Thr213, Pro156, His296, Phe266, I270, Cys113, Met326, Arg239, Pro328, Leu233, Phe219, Arg220, His193, Asn216 that are engaged with 6MSA moiety in a radius of 6 Å. The volume of active site cavity, as calculated by the program CASTp 3.0, was 934 Å3 [29]. Surface representation of residues forming the cavity in response to 6MSA-SNAC as shown in Figure S8.

To assess the binding specificity of ChlB3, a docking analysis was conducted with 6MSA-SNAC, acetyl-SNAC, and cyclohexyl-SNAC. As described previously, residues Cys113, His296 and Asp301 are conserved in ChlB3 and structurally related enzymes. Hence, docking analysis was done against the residue (Cys113), which exposed the binding affinity in terms of docking score of −4.2 kcal/mol, −7.7 kcal/mol, −5.7 kcal/mol and for acetyl-SNAC, 6MSA-SNAC, and cyclohexonyl-SNAC, respectively.

### 2.5. MD Simulation Confirms the 6MSA-SNAC Is Suitable Substrate

**Figure 6:**
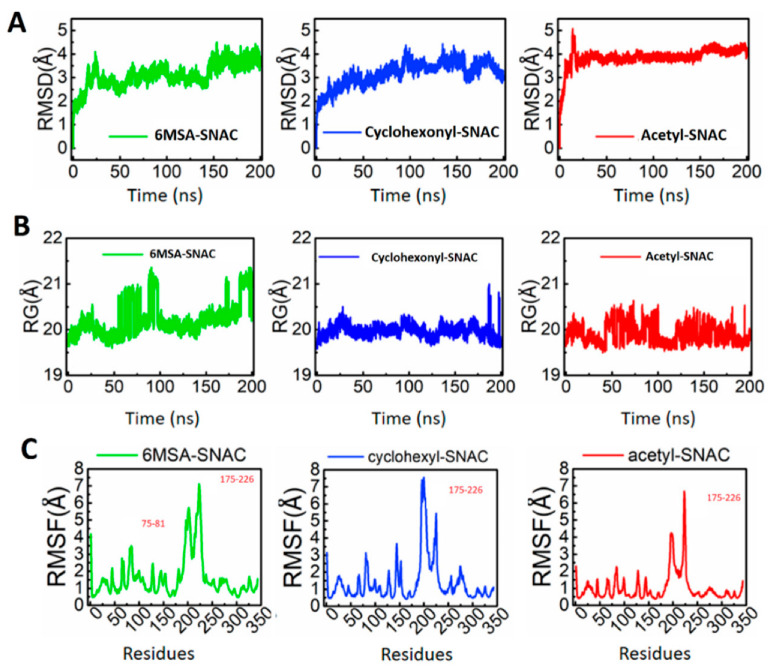
(**A**) Representation of the structural stability of the 6MSA-SNAC, cyclohexonyl-SNAC and acetyl-SNAC complexes, (**B**) The Rg graph of each complex. (**A**) 6MSA-SNAC; (**B**) acetyl-SNAC; (**C**) cyclohexonyl-SNAC. The x-axis shows the simulation time, while the y-axis shows Rg in Å. A 200 ns trajectory was used to calculate the compactness as Rg. (**C**) The RMSF graphs for each complex are shown in different colors. The x-axis shows the total number of residues while the y-axis shows RMSF in Å.

For system stability, the root mean square deviation (RMSD) of 6MSA-SNAC, acetyl-SNAC and cyclohexonyl-SNAC was calculated. For this purpose, docked substrates of 6MSA-SNAC, cyclohexonyl-SNAC and acetyl-SNAC were used (hereafter referred to by their short substrate names for clarity; see Table S2). The results showed that RMSD values of 6MSA-SNAC were quickly increased in the first 20 ns of simulation. These high RMSD values explain the encounter of substrate for dynamic environment to gain conformational changes suitable for substrate attachment. At 3.0 Å RMSD, the structure was stabilized, and deviation was observed at 50 ns, 100 ns and 150 ns of different time intervals. However, structure showed little fluctuation from mean position during the 150 ns simulation time (Figure 6A). These fluctuations in the complex system could be caused by changes in the characteristic motion of substrate in the protein binding site. Once stable conformation was accomplished, the system continued smoothly till 200 ns.

**Figure 7:**
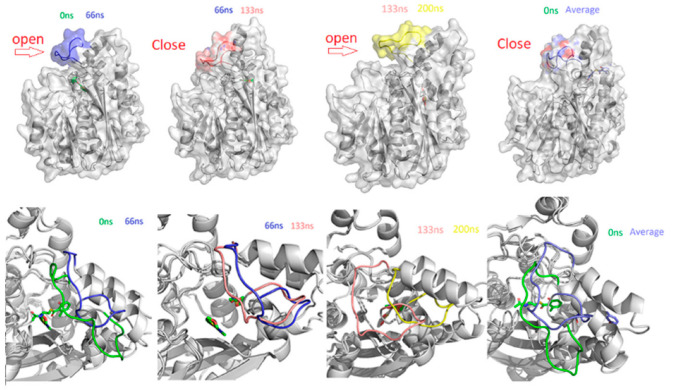
Represent the open and close state of protein in response to simulation. represent the variations captured during the simulation at different time intervals of 0 ns, 66 ns, 133 ns and 200 ns experienced by the 6MSA-SNAC complex. Each complex is shown in different colors. The substrate binding and the loop 196–211 are presented in different panels at a specific time point.

To explore fluctuations at specific time periods, we recovered structural frames of ChlB3 with 6MSA-SNAC, cyclohexonyl-SNAC and acetyl-SNAC complexes, and studied the molecular mechanism of substrate binding together with the effect of SNAC on the binding and stability of protein (Figure 7 and Figure S9). In 6MSA-complex, loop comprising residues (196–211) deviated from its original position and caused the conformational change to open binding cavity at 0 ns-66 ns. Similarly, opening of the loop was observed at 133 ns, allowing the substrate to move away from the active side of protein. Except for the time intervals stated, the substrate and loop are virtually in their original location in the average structure, demonstrating that substrate binding is stable (Figure 7).

Furthermore, when compare the all the simulated complex-structures, acetyl-SNAC displayed comparatively an unstable behavior throughout the simulation faced significant structural deviation (Figure 6).

We also calculated the structural compactness as the radius of gyration (Rg). In the case of the 6MSA-SNAC, the structure initially remained closed; however, after 20 ns the Rg value increased from 20 Å to 20.5 Å. This pattern was changed to unequal distribution of Rg until 100 ns and the average Rg was reported to be 22 Å. This sudden change in Rg with time apparently is due to the movement of substrate in and out of the active pocket, providing more space for free movement inside the pocket. However, the substrate remained inside the pocket for most of the simulation time. Unlike the 6MSA-SNAC, the acetyl-SNAC and cyclohexonyl-SNAC complexes remained compact. For acetyl-SNAC and cyclohexonyl-SNAC the calculated average Rg was 20 Å and 19.5 Å, respectively (Figure 6B).

To investigate the local flexibility of a protein complex, RMSFs value of backbone Cα was calculated for 6MSA-SNAC, acetyl-SNAC and cyclohexonyl-SNAC (Figure 6C). The higher RMSF value indicates flexible region with more movements, whereas the low RMSF value suggests a rigid region resulted in minimal movements during the simulation. As shown in Figure 6C, all the complexes with SNAC i.e., 6MSA-SNAC, acetyl-SNAC and cyclohexonyl-SNAC exhibit a more similar pattern of minimal residual flexibility except higher flexibility in some regions, i.e., 75–81 and 175–226. Furthermore, these flexible regions are responsible for overall structural deviation in initial coordinates.

**Figure 8:**
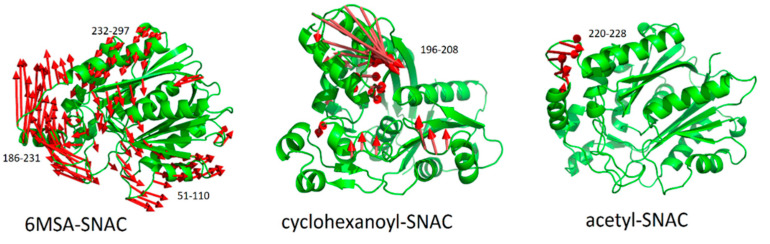
PCA analysis showing the magnitude of dominant movements of protein regions for 6MSA-SNAC, acetyl-SNAC and cyclohexonyl-SNAC complexes calculated from simulated trajectories.

To understand the dynamics function relationship as a protein motion, principal component analysis (PCA) was determined. Analysis revealed that 6MSA-SNAC complex showed more dominant movements as compared to the other two complexes (Figure 8). The residues 186–231 showed clockwise rotation in the opposite direction from residues 51–110 and 232–297. The later residues showed anti-clockwise rotation away from the adjacent residues. These findings demonstrated that the ChlB3 enzyme underwent large-scale conformational changes during the reaction process to accommodate the 6MSA-SNAC substrate. On the other hand, the mobility of the above-mentioned residues in cyclohexonyl-SNAC and acetyl-SNAC complexes are not much significant. However, the cyclohexonyl-SNAC complex showed prominent movements only in loop reign of residues 196–208, while the acetyl-SNAC complex showed slight movements in loop region residues 220–228. These alterations elucidated how the substrate triggers one enzyme from a closed (inactive) conformation to open (active) conformation. The observed motions might be responsible for activation of the enzyme for the particular substrates. When the contacts of the substrates with the pocket residues were calculated (see Figure S10), we discovered that the 6MSA-SNAC form high contacts (ranges from 38% to 80% of the total population) with neighboring residues while acetyl-SNAC form the least contacts (<30%). The contacts for cyclohexonyl-SNAC come in between these two extremes (12% to 45%). All together, these results illustrate that substrate-pocket interaction are very important for the overall motions observed in PCA analysis that push the enzyme from static to a functional state.

## 3. Materials and Methods

### 3.1. Protein Expression and Purification

Using a standard protocol, a fragment encoding ChlB3 was amplified from *Streptomyces antibioticus* DSM-40725 genomic DNA [30,31]. The primers used for ChlB3 amplification are listed in Table S1. *chlB3* comprising 1044 bp was cloned into pET28a with *Nhe*I and *Hind*III restriction enzymes. For gene expression, a plasmid containing *chlB3* was transformed into *Escherichia-coli* BL21 (DE3). Transformed cells were cultured at 37 °C in 6L Luria-Bertani (LB) medium supplemented with 50 mg kanamycin. The cells were cultured to 0.4 at OD 600 and induced with 0.3 mM Isopropyl β-D-1-thiogalactopyranoside (IPTG) at 16 °C for 20 h. For cell collection, the culture was centrifuged at 5000× *g* for 15 min, resuspended in the lysis buffer (50 mM Tris, 400 mM NaCl, 5% glycerol, pH 7.5), and sonicated at 4 °C for 12 min. The samples were again subjected to centrifugation (35 min at 20,000× *g*) to remove the cell debris. Finally, the supernatant was loaded onto Nickel-NTA resin equilibrated with lysis buffer. The samples were cleaned by lysis buffer with imidazole of 10 mM (pH 7.5). Further, the lysis buffer comprising 300 mM imidazole was used to elute the His-tagged protein. The eluted protein was polished using a Superdex-200 gel filtration column (GE Healthcare), equilibrated with a buffer containing 10 mM Tris (pH 7.5), 150 mM NaCl, and 10% *v/v* glycerol. The resultant protein was concentrated to 10.5 mg/mL and exchanged with buffer containing 10 mM Tris (pH 7.5), 25 mM NaCl, and 10% (*v/v*) glycerol. Using a size exclusion column, purified ChlB3 protein was observed with a molecular weight of ~38 kDa (Figure S1).

### 3.2. Kinetic Analyis

The Ellman’s reagent [5,5-dithio-bis-(2-nitrobenzoic acid)-DTNB] was used to examine the free thiol group (SH) of the released SNAC [32]. Several control reactions were carried out in parallel to remove the absorbance triggered by cysteine residues in the ChlB3 enzyme. All the reactions were examined in Tris buffer (100 μL, 50 mM, pH 7.5) containing 1 μM ChlB3 and 5 μM–3 mM substrate (6MSA-SNAC, acetyl-SNAC, cyclohexonyl–SNAC) at room temperature. To stop the reaction, a similar volume of dimethyl sulfoxide was added, and aliquots were removed. After the addition of DTNB (1mM), the samples were incubated for 10 min. The release of SNAC catalyzed by the enzyme of respective substrates was analyzed spectrophotometrically at 412 nm. The samples were tested in triplicates and the kinetic parameters were derived by nonlinear regression analysis based on Michaelis−Menten kinetics using OriginLab^®^ software (Version 9.0). All the R-SNAC thioesters used for functional assays were synthesized by a similar protocol as explained by Wang et al., and Abugrain et al. [27,33].

### 3.3. Chromatographic Analysis

To analyze the hydrolytic activity, ChlB3 (5 μM) was incubated with 1 mM 6MSA-SNAC in 100 μL of Tris buffer (pH 7.5). The reaction was quenched after 12 h with equal volume of methanol and centrifuged at 6000× *g* for 1 min. The resulting supernatant was subjected to chromatographic analysis. For high performance liquid chromatography (HPLC), 20 µL supernatant was injected into a reversed phase column (C18; Zorbax 300 SB, 4.6 × 250 mm; 5 micron). The peaks were observed at 245 nm wavelength using mobile phase i.e., 100 H_2_O to 100 % methanol for 30 min. For LC/MS analysis, Ultra-Performance Liquid Chromatography (LC/TOF/MS-Waters) was used. The mobile phase (A: water containing 0.1% formic acid, B: 100% methanol) was run using a C18 column at 0.4 mL/min flow rate. At room temperature, the mobile phase gradient was: 50–90% B solution, 10–12 min followed by 90% B solution, 12–15 min. The data and the corresponding peaks were evaluated by using UNIFI 1.8.

### 3.4. Crystallization and Structure Determination

Crystals of ChlB3 were grown by the sitting drops vapor diffusion method, consisting of 1.5 μL protein stock solution and 0.5 μL precipitant solution (0.1 M HEPS pH 7.0, 1.4 M sodium acetate-trihydrate). The crystals were obtained in three days at 20°C. The resulting crystals were soaked in 25% (*v/v*) glycerol for 10 s and frozen in liquid nitrogen. The diffraction data were collected from beamlines BL19U at Shanghai Synchrotron Radiation Facility and processed with HKL3000 [34]. The structure was solved by molecular replacement using 4XSA as a search model and refined to 3.1 Å resolution at R_work_ and R_free_ values of 0.226 and 0.256, respectively (Table 1). The structure was refined by refmac, built by coot and visualized by PyMOL [35,36,37]. The atomic coordinates and structure factor amplitudes have been deposited in the Protein Data Bank (PDB) under accession code 7EQI. Further details are present under uniport Id Q0R4P5.

### 3.5. Docking Analysis

Auto-dock-vina 3.1 was used to prepare the 6MSA-SNAC and ChlB3 complex for MD simulation [38]. Ligands were docked to the ChlB3 structure in a grid box centered on catalytic Cys113. From docking results, seven different possible binding modes were obtained, and the modes with minimum energy were selected.

### 3.6. Molecular Dynamic Simulation

The biomolecular simulation program AMBER Tools version 14 was used for the execution of efficient simulations with periodic boundary conditions [39]. The AMBER ff14SB force field was used for topology and initial coordinates generation. For optimization, i.e., solvation neutralization and protonation, LEaP module of AMBER14 was used [39,40]. For systems solvation, TIP3P water octahedral box was utilized. Direct Coulomb and van der Waals interactions were computed with 8 Å distance. The mesh Ewald method was used to resolved the long range electrostatic interactions [41]. Production and preparation runs were executed using Langevin thermostat along with friction constant of 1 ps^−1^ and Berendsen thermostat, respectively [42].To perform the simulation, NVIDIA^®^ Tesla K40CUDA, GPU was employed. For the structure optimization in a solvated system two steps of minimization, the macromolecules frozen 500-step steepest descent minimization and 2000-step conjugate gradient minimization were performed, respectively. Further, the complete system was subjected to 1000 steps descent minimization and 19,000 steps conjugate gradient minimization. System was equilibrated and heated in NVT at 310 K with respective time of 200 ps and 400 ps. After heating and equilibration, MD production was performed once for each system at constant pressure and temperature (NPT) ensemble. Time-scale simulations of 200 ns were sufficient for the system to reach an equilibrium state. The simulation details are given in Supplementary Table S2.

## 4. Conclusions

In conclusion, understanding the molecular basis for ChlB3 selectivity is critical for incorporating unnatural substrates into polyketide products. Interestingly, ChlB3 hydrolyzed a number of thioester compounds with a relatively relaxed substrate tolerance. Furthermore, X-ray crystal structures solved at 3.1 Å resolution revealed that ChlB3 adopts KSIII-like fold to work as acyltransferase. In addition, RMSD, RMSF, and PCA proved the flexibility and stability of the simulated trajectories. The structural and functional analyses of ChlB3 described here, in conjunction with computational analysis, will aid in understanding the catalytic mechanism for thioester compounds.

## Figures and Tables

**Table 1:** Crystallographic data collection and refinement statistics. Parentheses indicate values for the shell with the highest resolution.

Data Collection	ChlB3
Wavelength (Å)	0.9793
Space group	P2_1_2_1_2_1_
*α*, *b*, *c* (Å)	99.473 186.583 189.750
Resolution (Å)	50–3.1
*R*_merge_	0.115 (0.589)
I/σ I	14.9 (3.7)
CC_1/2_	0.884
Completeness (%)	99.5 (99.9)
Redundancy	6.0 (5.9)
Refinement statistics	
Resolution (Å)	50–3.1
Unique reflections	74,294
*R*_work_ */R*_free_	0.226/0.256
No. of atoms	
Protein	19057
*B* factor	
Protein	67
R.m.s.d. (Å)	
Bond lengths (Å)	0.01
Bond angles (^o^)	1.6

## Data Availability

The atomic coordinates and structure factor amplitudes have been deposited in the Protein Data Bank (PDB) under accession codes 7EQI.

## Supplementary Materials

The following supporting information can be downloaded at: https://www.mdpi.com/article/10.3390/molecules27196405/s1, Figure S1. Molecular weight determined by size-exclusion chromatography. (A) The uncropped SDSPAGE of ChlB3. (x indicated irrelevant sample, ChlB3 protein was present on right column). (B) A standard curve of the log (Molecular weight) versus Kav was generated using the Protein standard. ChlB3 migrates at ~38 kDa (C) Size exclusion chromatogram of ChlB3. Figure S2. Ramachandran plot of ChlB3. Details: 93.2% (2421/2599) of all residues were in favored (98%) regions. 99.2% (2579/2599) of all residues were in allowed (>99.8%) regions. The left most (General case) panel represent the Φ and Ψ distribution for all residues (excluding Isoleucine and Valine, pre-Proline, Glycine, trans-Proline, cise-Proline residues). The rest of the panels represent the Φ and Ψ data points for individual residues Isoleucine-Valine, pre-Proline, Glycine, trans-Proline, cise-Proline, as these residues are significantly different from the other amino acids in their backbone stereochemistry. There were 20 outliers (phi, psi): A 99 Leu (−162.5, −73.0): A 195 Gly (−179.4, 79.2) B 47 Gly (−71.0, −90.1): B 146 Thr (61.7, −177.2): B 147 Pro (−61.4, −49.9): C 147 Pro (−81.3, −54.5): D 99 Leu (−163.6, −68.6): D 132 Arg (57.3, −144.3): D 204 Ala (−161.5, −97.2): E 47 Gly (−71.6, −92.0): E 99 Leu (−163.7, −74.5): E 131 Pro (−77.9, −154.7): E 132 Arg (75.1, −161.5): E 147 Pro (−109.2, −85.5): E 207 Gly (−31.0, 90.0): F 206 Ile (−171.7, 109.5). Figure S3. Structural comparison of substrate binding sites of Doxorubicin synthase-DpsC, CerJ and ChlB3. Light gray color represents the surface area of substrate binding pocket. Whereas Blue color represents the surface area of rest of the protein. (A) The substrate and the pocket of Doxorubicin synthase. (B) The substrate and the pocket of CerJ. (C) Binding surface representations of ChlB3. Figure S4. Phylogenetic tree of ChlB3 relevant to structurally related enzymes FabH, KSIII and CerJ. Tree was constructed using minimum evolution method, bootstrap 1000, with the alignment based on amino acids by MEGA X. Figure S5. Multiple alignment of ChlB3 with structurally related proteins, CerJ (PDB code-3S3L, 0.84 Å RMSD, 32.48% sequence identity) and Doxorubicin DpsC (5WGC, 0.6 Å RMSD, 34.50% sequence identity). Highly conserved regions are enclosed with boxes. Residues responsible for catalytic activity are indicated with *. Figure S6. (A) Hydrolytic reaction of ChlB3 in response to cyclohexanoyl-SNAC and acetyl-SNAC. Michaelis-Menten curves of ChlB3 for (B) cyclohexanoyl-SNAC (C) 6MSA-SNAC, and (D) Acetyl-SNAC. Figure S7. (A). Snapshot of average structure extracted from 200ns of MD simulation. Active site residues of ChlB3 form hydrogen bond interaction (represented in yellow lines) with 6MSA-SNAC depicted in stick. (B) Electrostatic view of ChlB3 with 6MSA-SNAC complex. Binding pocket is highlighted in blue color. Figure S8. Surface representation of residues surrounding 6MSA-SNAC. Figure S9. Structural stability of the Acetyl-SNAC and cyclohexonyl-SNAC complex. Different captured during the simulation at different time intervals 0 ns, 66 ns, 133 ns and 200 ns experienced by (A) acetyl-SNAC and (B) cyclohexonyl-SNAC complexes. The substrate binding and the loop 196-211 are presented in different panels at a specific time point. Figure S10. Contacts of SNAC substrate with pocket residues for 6MSA-SNAC, acetyl-SNAC and cyclohexonyl-SNAC complexes calculated from simulated trajectories. Table S1. Primer used for amplification of ChlB3. Table S2. Simulation details for three systems. ff14SB force field with TIP3P water model was used.
