# Supplementary material for: Structural Insight of KSIII (β-Ketoacyl-ACP Synthase)-like Acyltransferase ChlB3 in the Biosynthesis of Chlorothricin
Source: Molecules. 2022 Sep 28;27(19):6405. doi: 10.3390/molecules27196405 (PMC9573744; doi:10.3390/molecules27196405)
Supplement: Supplementary file 1 [file molecules-27-06405-s001.zip › molecules-1879271-supplementary.pdf]

Structural Insight of KSIII (β-ketoacyl-ACP synthase)-like acyltransferase ChlB3 in the Biosynthesis of Chlorothricin

Asad Ullah Saeed [1], Mueed Ur Rahman [2], Hai-Feng Chen [2,3] and Jianting Zheng [1,4,*]

[1] State Key Laboratory of Microbial Metabolism and School of Life Sciences and Biotechnology, Shanghai Jiao Tong University, Shanghai 200240, China

[2] State Key Laboratory of Microbial Metabolism, Joint International Research Laboratory of Metabolic & Developmental Sciences, Department of Bioinformatics and Biostatistics, National Experimental Teaching Center for Life Sciences and Biotechnology, School of Life Sciences and Biotechnology, Shanghai Jiao Tong University, Shanghai 200240, China

[3] Shanghai Center for Bioinformation Technology, Shanghai 200235, China

[4] Joint International Research Laboratory of Metabolic & Developmental Sciences, Shanghai Jiao Tong University, Shanghai 200240, China

* Correspondence: jtzheng@sjtu.edu.cn

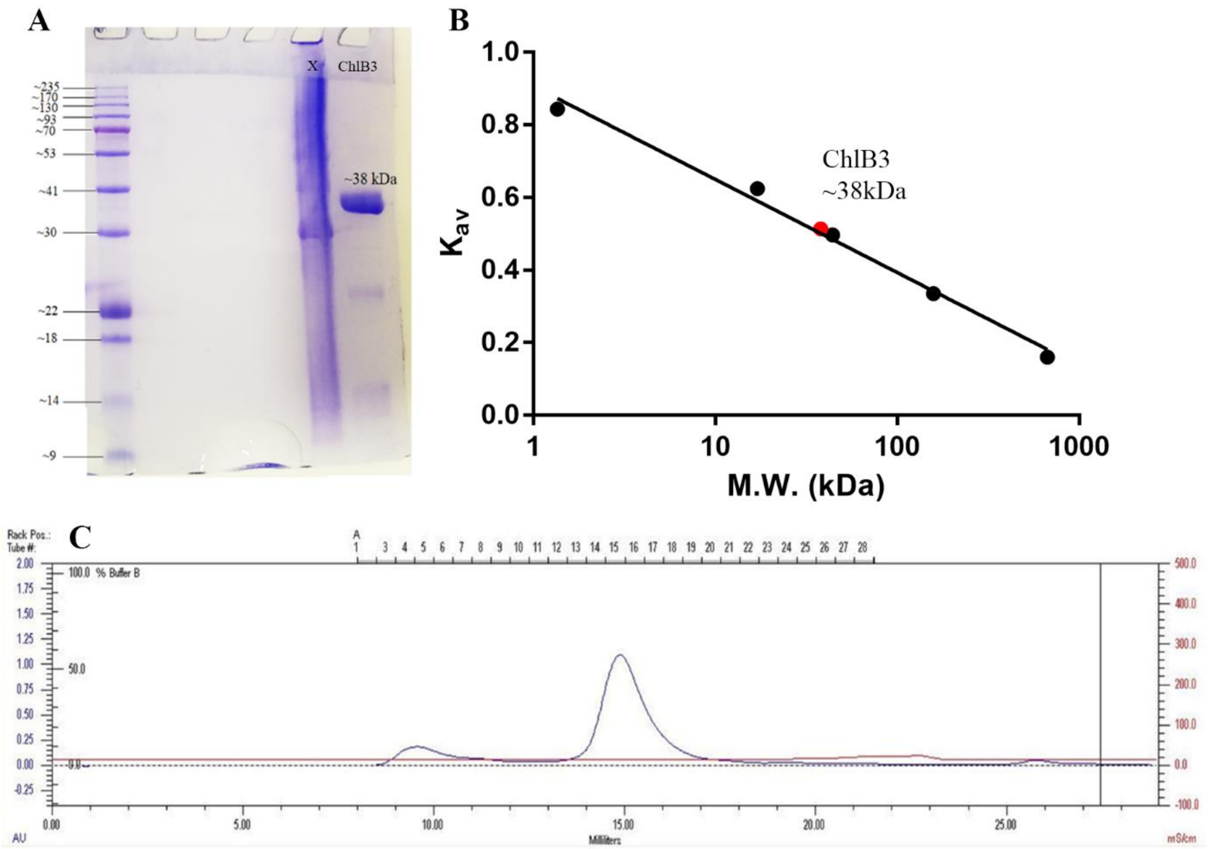

Figure S1. Molecular weight determined by size-exclusion chromatography. (A) The uncropped SDS-PAGE of ChlB3. (x indicated irrelevant sample, ChlB3 protein was present on right column). (B) A standard curve of the log (Molecular weight) versus Kav was generated using the Protein standard. ChlB3 migrates at ~38 kDa (C) Size exclusion chromatogram of ChlB3

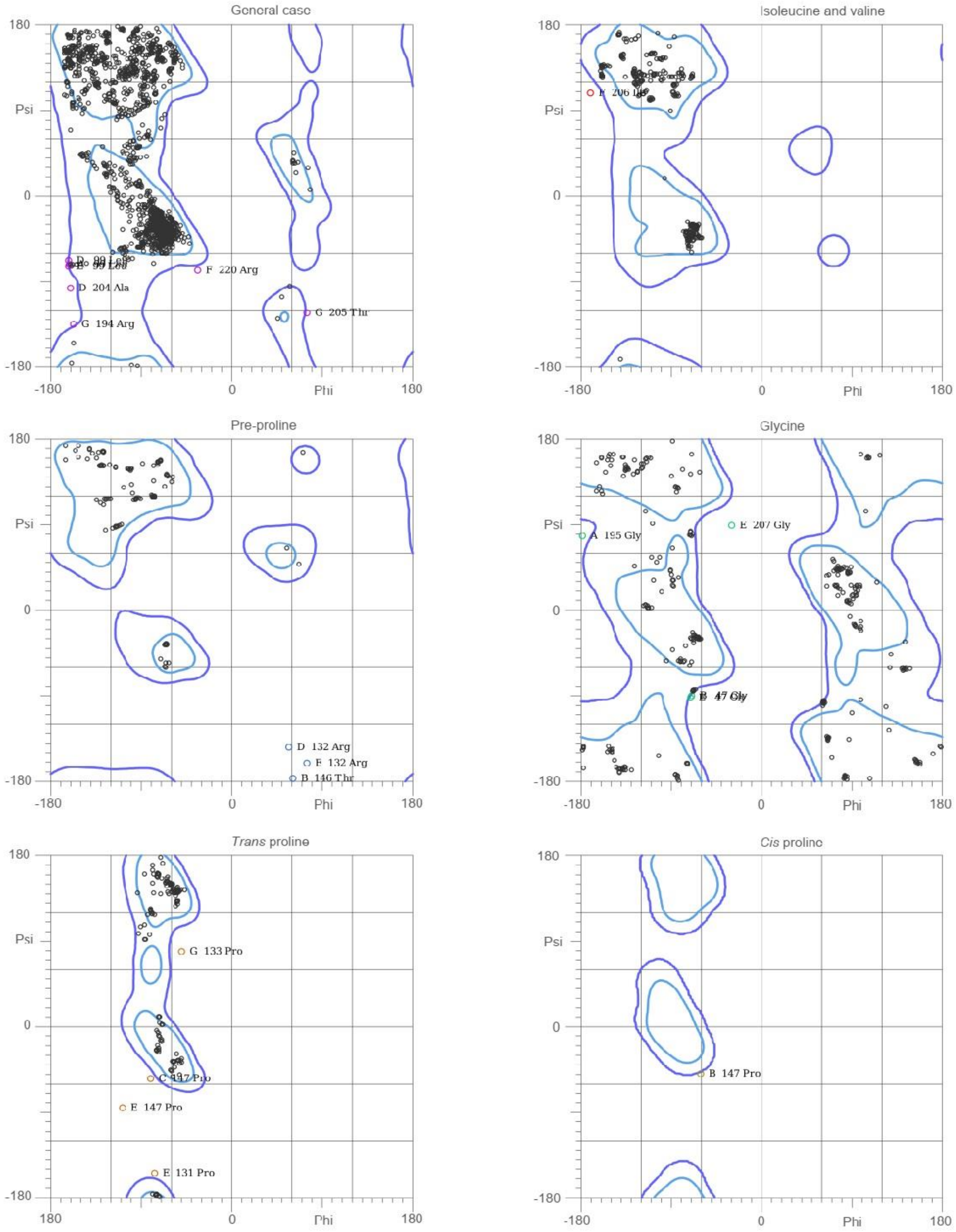

Figure S2. Ramachandran plot of ChlB3. Details: 93.2% (2421/2599) of all residues were in favored (98%) regions. 99.2% (2579/2599) of all residues were in allowed (>99.8%) regions. The left most (General case) panel represent the Φ and Ψ distribution for all residues (excluding Isoleucine and Valine, pre-Proline, Glycine, trans-Proline, cise-Proline residues). The rest of the panels represent the Φ and Ψ data points for individual residues Isoleucine-Valine, pre-Proline, Glycine, trans-Proline, cise-Proline, as these residues are significantly different from the other amino acids in their backbone stereochemistry. There were 20 outliers

(phi, psi): A 99 Leu (-162.5, -73.0): A 195 Gly (-179.4, 79.2) B 47 Gly (-71.0, -90.1) : B 146 Thr (61.7, -177.2) : B 147 Pro (-61.4, -49.9): C 147 Pro (-81.3, -54.5) : D 99 Leu (-163.6, -68.6) : D 132 Arg (57.3, -144.3) : D 204 Ala (-161.5, -97.2) : E 47 Gly (-71.6, -92.0) : E 99 Leu (-163.7, -74.5) : E 131 Pro (-77.9, -154.7) : E 132 Arg (75.1, -161.5): E 147 Pro (-109.2, -85.5) : E 207 Gly (-31.0, 90.0) : F 206 Ile (-171.7, 109.5)

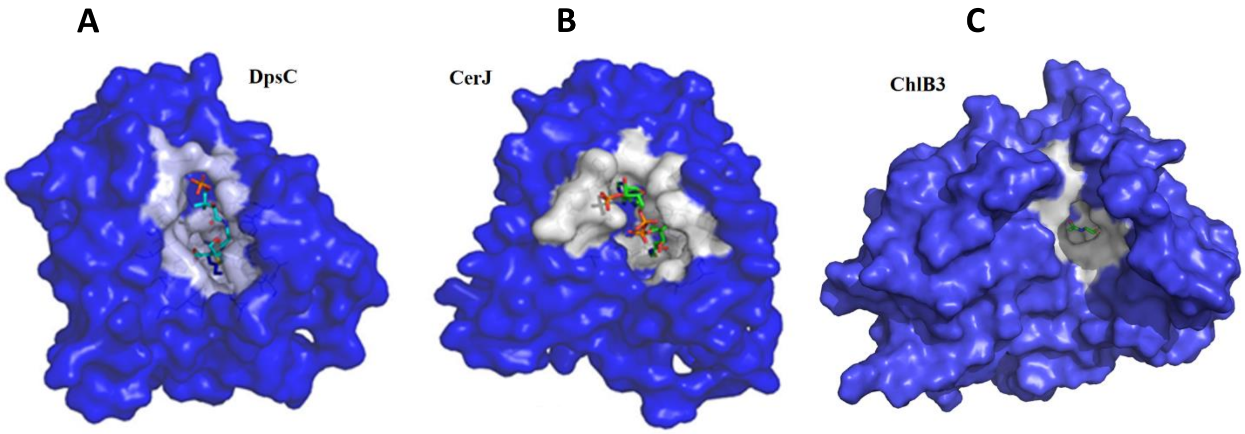

Figure S3. Structural comparison of substrate binding sites of Doxorubicin synthase-DpsC, CerJ and ChlB3. Light gray color represents the surface area of substrate binding pocket. Whereas Blue color represents the surface area of rest of the protein. (A) The substrate and the pocket of Doxorubicin synthase. (B) The substrate and the pocket of CerJ. (C) Binding surface representations of ChlB3

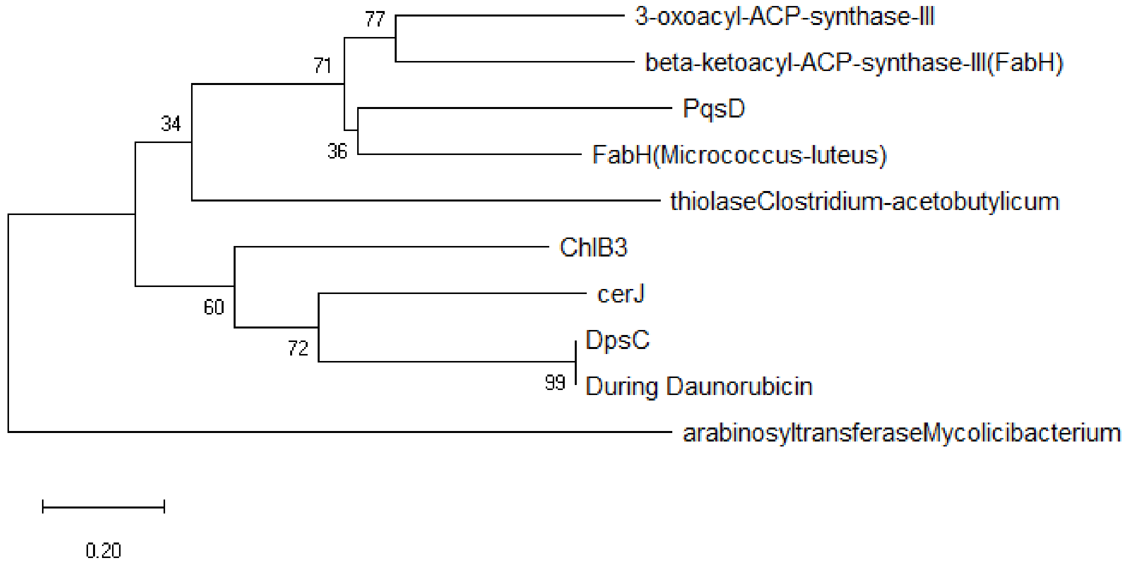

Figure S4. Phylogenetic tree of ChlB3 relevant to structurally related enzymes FabH, KSIII and CerJ. Tree was constructed using minimum evolution method, bootstrap 1000, with the alignment based on amino acids by MEGA X.

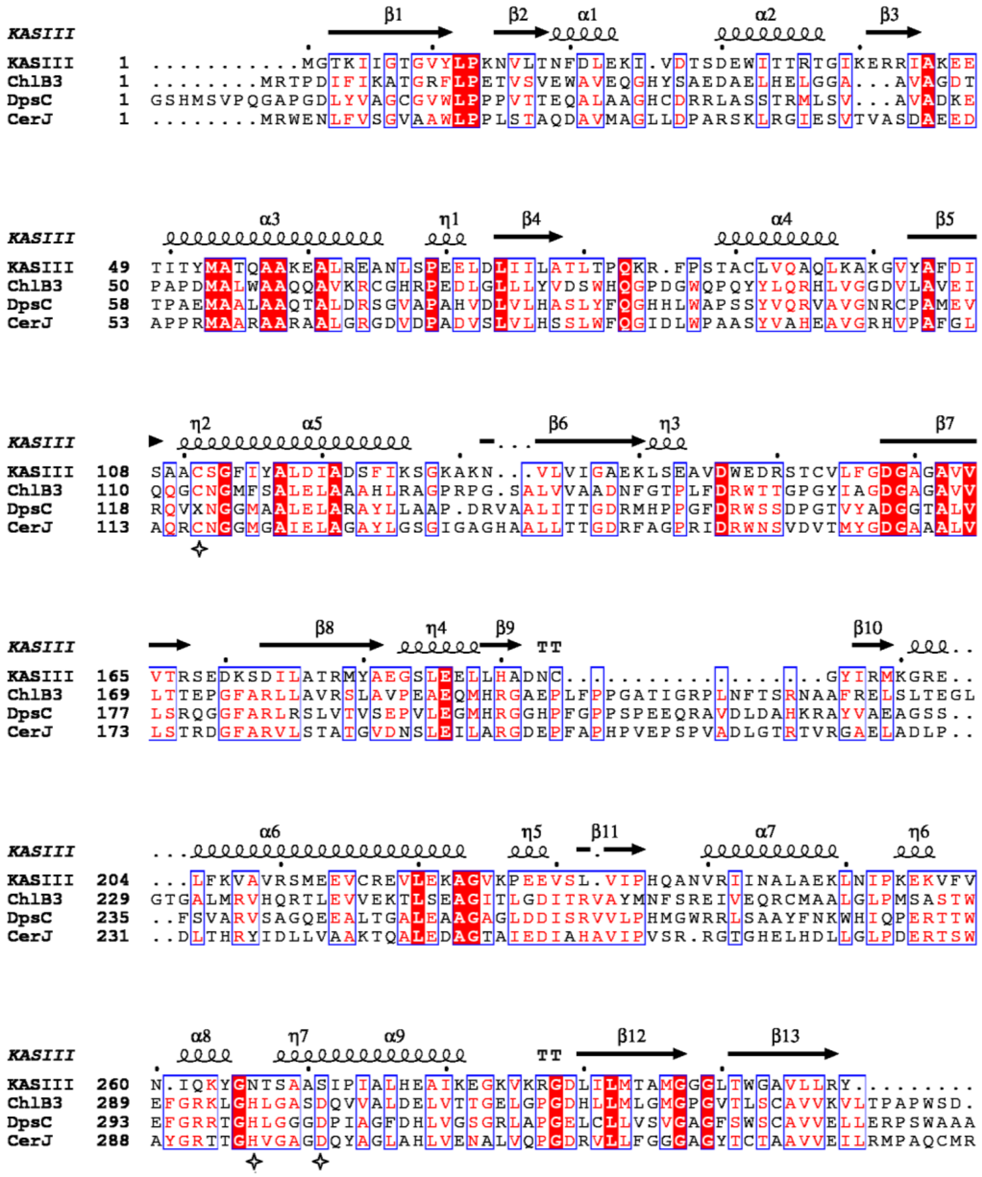

Figure S5. Multiple alignment of ChlB3 with structurally related proteins, CerJ (PDB code–3S3L, 0.84 Å RMSD, 32.48% sequence identity) and Doxorubicin DpsC (5WGC, 0.6 Å RMSD, 34.50% sequence identity). Highly conserved regions are enclosed with boxes. Residues responsible for catalytic activity are indicated with *.

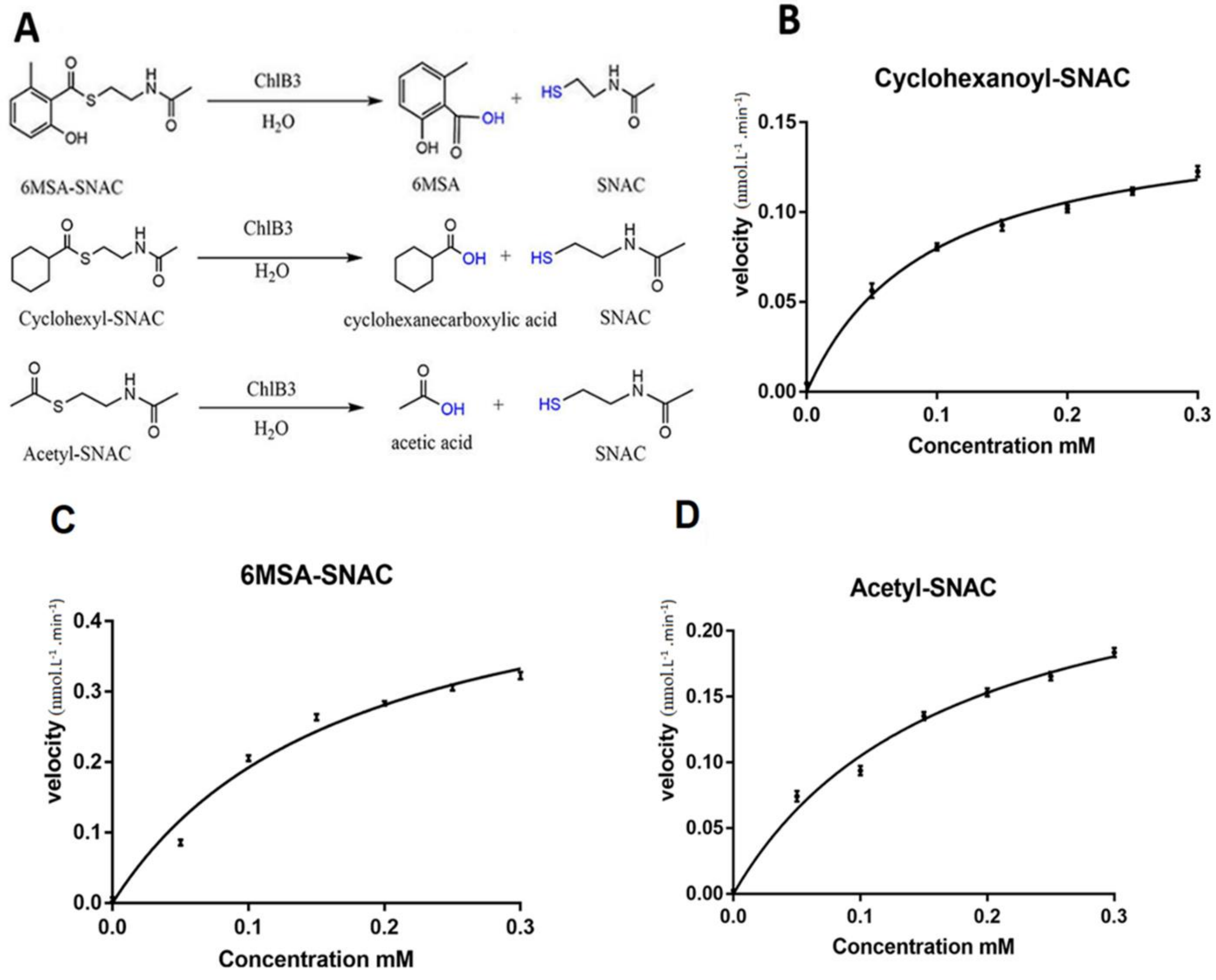

Figure S6. (A) Hydrolytic reaction of ChlB3 in response to cyclohexanoyl-SNAC and acetyl-SNAC. Michaelis-Menten curves of ChlB3 for (B) cyclohexanoyl-SNAC (C) 6MSA-SNAC, and (D) Acetyl-SNAC.

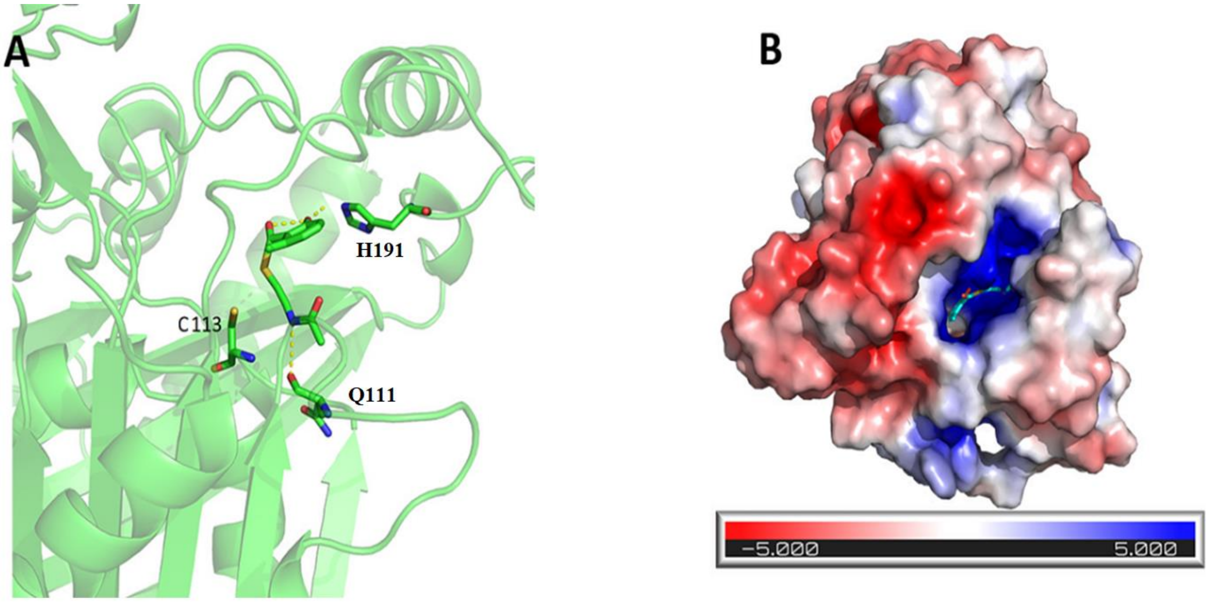

Figure S7. (A). Snapshot of average structure extracted from 200ns of MD simulation. Active site residues of ChlB3 form hydrogen bond interaction (represented in yellow lines) with 6MSA-SNAC depicted in stick. (B) Electrostatic view of ChlB3 with 6MSA-SNAC complex. Binding pocket is highlighted in blue color.

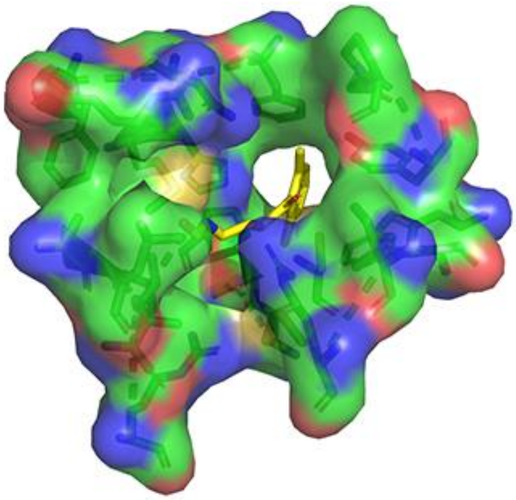

Figure S8. Surface representation of residues surrounding 6MSA-SNAC.

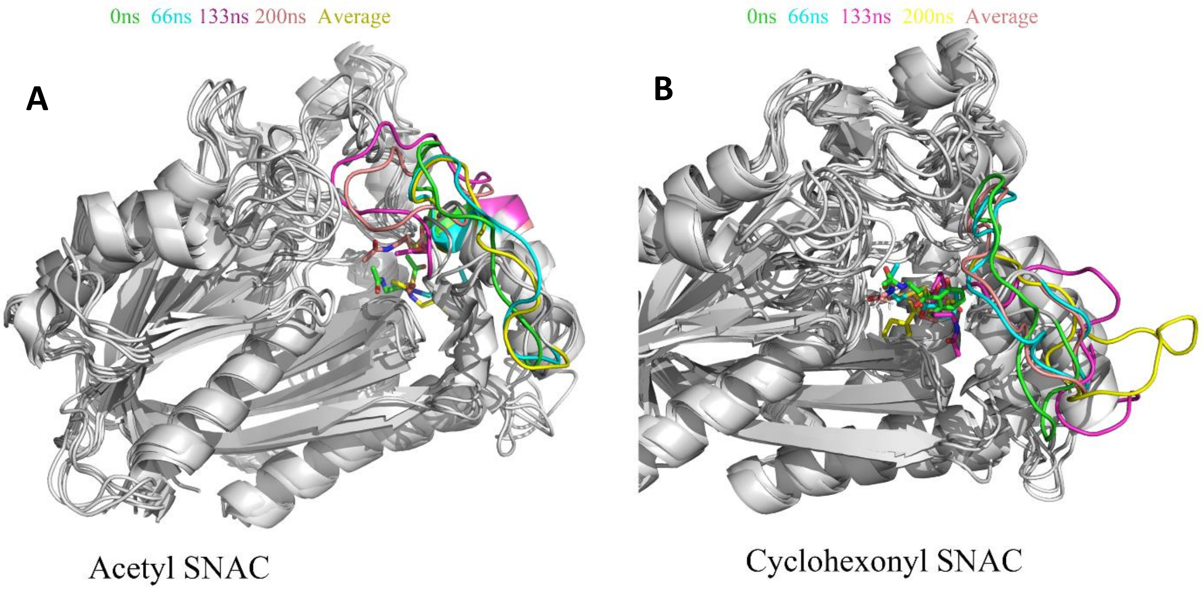

Figure S9. Structural stability of the Acetyl-SNAC and cyclohexonyl-SNAC complex. Different captured during the simulation at different time intervals 0 ns, 66 ns, 133 ns and 200 ns experienced by (A) acetyl-SNAC and (B) cyclohexonyl-SNAC complexes. The substrate binding and the loop 196-211 are presented in different panels at a specific time point.

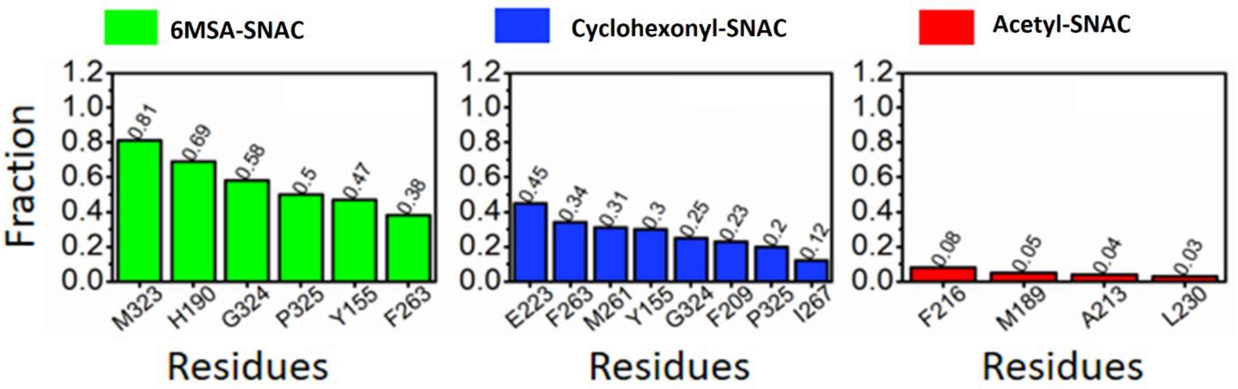

Figure S10. Contacts of SNAC substrate with pocket residues for 6MSA-SNAC, acetyl-SNAC and cyclohexonyl-SNAC complexes calculated from simulated trajectories.

Table S1. Primer used for amplification of ChlB3.

Primer ID	Sequence
ChlB3_ Forward Primer	atcgtaatgctagcgtgcggacgcccgacatattcatc
ChlB3_ Reverse Primer	tgattcgataagctttcagtcgctccagggagccggcgt

Table S2. Simulation details for three systems. ff14SB force field with TIP3P water model was used.

System Name	Substrate name	System Composition	Time (ns)	Temperature (K)
ChlB3-6MSA-SNAC	6MSA-SNAC	6-methylsalicylic acid + ChlB3	200	310
ChlB3-ACE-SNAC	acetyl-SNAC	Acetyl-SNAC + ChlB3	200	310
ChlB3-CLH-SNAC	cyclohexonyl-SNAC	Cyclohexonyl-SNAC + ChlB3	200	310
